# Long non-coding RNA RP11-468E2.5 curtails colorectal cancer cell proliferation and stimulates apoptosis via the JAK/STAT signaling pathway by targeting STAT5 and STAT6

**DOI:** 10.1186/s13046-019-1428-0

**Published:** 2019-11-12

**Authors:** Li Jiang, Xu-Hai Zhao, Yin-Ling Mao, Jun-Feng Wang, Hui-Jun Zheng, Qing-Shan You

**Affiliations:** 1 Department of Hematology and Lymphatic Diseases, Harbin Medical University Tumour Hospital, Harbin, 150081 People’s Republic of China; 2 Department of Breast Surgery, Harbin Medical University Tumour Hospital, Harbin, 150081 People’s Republic of China; 3 Department of Abdominal Radiotherapy, Harbin Medical University Tumour Hospital, No. 150, Haping Road, Nangang District, Harbin, 150081 People’s Republic of China; 4 Department of Thoracic Surgery, Harbin Medical University Tumour Hospital, Harbin, 150081 People’s Republic of China; 5 Department of General Surgery, Kangying Hospital of Mingshui County, Suihua, 151700 People’s Republic of China

**Keywords:** Long non-coding RNA RP11-468E2.5, Colorectal cancer, STAT5 gene, STAT6 gene, Janus kinase-signal transducer and activator of transcription signaling pathway, Proliferation, Apoptosis

## Abstract

**Background:**

Long non-coding RNAs (lncRNAs) are tumor-associated biological molecules and have been found to be implicated in the progression of colorectal cancer (CRC). This study aims to examine the effects of lncRNA RP11-468E2.5 and its target genes (STAT5 and STAT6) on the biological activities of CRC cells via the Janus kinase-signal transducer and activator of transcription (JAK/STAT) signaling pathway.

**Methods:**

We initially screened the GEO database for differentially expressed lncRNAs related to CRC and then made a prediction of the implicated target genes. Then we collected CRC tissues and adjacent normal tissues from 169 CRC patients. Human CRC HCT116 and SW480 cells were treated with small interference RNA (siRNA) against RP11-468E2.5, AG490 (an inhibitor of the JAK/STAT signaling pathway), or both in combination. Next, we measured the effects of RP11-468E2.5 treatment on cellular activities such as cell viability, cycle distribution and cell apoptosis, and studied interactions among RP11-468E2.5, STAT5/STAT6, and the JAK/STAT signaling pathway. Finally, an in vivo tumor formation assay was performed to observe the effect of RP11-468E2.5 on tumor growth.

**Results:**

The CRC-related gene microarray data showed low expression of RP11-468E2.5 in CRC surgical specimens. However, RP11-468E2.5 was confirmed to target STAT5 and STAT6, which participate in the JAK/STAT signaling pathway. CRC tissues showed lower expression of RP11-468E2.5, higher expression of STAT5, STAT6 and of the cell cycle marker Cyclin D1 (CCND1), compared to the findings in adjacent normal tissues. The treatment of siRNA against RP11-468E2.5 increased expression of JAK2, STAT3, STAT5, STAT6, CCND1 and Bcl-2 along with the extent of STAT3, STAT5 and STAT6 phosphorylation, while lowering expression of P21 and P27. Treatment with AG490 exhibited approximately opposite effects, whereas siRNA against RP11-468E2.5 treatment stimulated CRC cell proliferation and reduced cell apoptosis, while promoting cell cycle entry; AG490 treatment reversed these results.

**Conclusions:**

Altogether, we conclude that up-regulation of RP11-468E2.5 inhibits the JAK/STAT signaling pathway by targeting STAT5 and STAT6, thereby suppressing cell proliferation and promoting cell apoptosis in CRC.

## Background

Colorectal cancer (CRC) is an aggressive disease with high morbidity and mortality throughout the world [1]. Each year, more than 1 million people are affected by CRC, accompanied by overt metastatic or invasive disease. The malignant form of CRC accounts for some 600,000 deaths worldwide each year [2]. Aging, mutations, and chronic intestinal inflammation are all known factors responsible for the occurrence and progression of CRC [3]. The high rates of cancer metastasis, recurrence and emergent chemoresistance pose great obstacles to effective treatments of patients with CRC at all stages, highlighting the need for the novel improved therapeutic strategies [4].

Long non-coding RNAs (lncRNAs) have been shown to play a crucial role in the regulation of tumorigenesis, and molecular biology studies implicate abnormal expression levels of lncRNAs such as LINC00152 in the development and progression of CRC cell tumorigenesis [5]. LncRNAs also serve as regulators of gene expression in interaction with diverse mechanisms. Regulation by lncRNAs depends on its site-specific interaction with DNA, as well as on their binding to proteins and chromosomes forming protein complexes [6]. Janus kinase-signal transducer and activator of transcription (JAK/STAT) signaling pathway is considered an important signal transduction pathway for cell development [7]. Previous studies have revealed that phosphorylated and non-phosphorylated STAT proteins are constitutively present in cytoplasm and nuclei. Other studies also proved that the dimer of phosphorylated STAT forms in the cytoplasm and then migrates into the nucleus. Only phosphorylated STAT homodimer or heterodimer species possess a DNA-binding capability. Upon combination with co-activator proteins, these species mediate transcriptional regulation [8, 9]. Under stimulation from cytokines, the messenger signal transducer and activator of transcription-5 tyrosine phosphorylation (pY-STAT5) are transiently activated, whereas STAT5 and the promoted pY-STAT5 show persistent overexpression in multiple neoplastic cell types [10]. Moreover, there is reportedly an underlying biological interaction between different STATs, i.e. STAT5 and STAT6. This pair of proteins functions as an activator and inhibitor for gene expression, as well as a modulator of the epigenetic landscape of immune cells [11].

A previous report indicated a positive correlation between the activation of the JAK/STAT signaling pathway and colorectal adenoma progression [12]. Another previous study suggested a relationship between lncRNAs and the JAK/STAT signaling pathway, which indicated a regulatory potential in biological processes [13]. Furthermore, Mao et al. have shown that elevated phospho-STAT5 expression is prevalent in adenocarcinoma of the colon and is associated with poor prognosis [14, 15]. Therefore, this present study aims to investigate the role of lncRNA RP11-468E2.5 on proliferation and apoptosis of CRC cells via interaction with the JAK/STAT signaling pathway and STAT5 and STAT6.

## Materials and methods

### Ethics statement

This study was performed with the approval from the Ethics Committee of the Harbin Medical University Tumour Hospital. All participating patients provided written informed consents. Animal experiments in this study were carried out in strict accordance with the Guide for the Care and Use of Laboratory animals published by the US National Institutes of Health.

### Microarray-based gene expression profiling

The Gene Expression Omnibus (GEO) database (http://www.ncbi.nlm.nih.gov/geo) was used to download CRC-related microarray expression data (GSE4107 and GSE21510) and annotate probe files. Background correction and normalization of each data microarray were processed by the Affy installation package of R software [16]. The empirical Baysian method of the linear model in the Limma installation package was combined with a traditional *t*-test to filter out nonspecific expression data, and then screen out the differentially expressed lncRNAs [17], which were predicted by the Multi Experiment Matrix website (MEM, http://biit.cs.ut.ee/mem/). The target gene was analyzed by Kyoto Encyclopedia of Genes and Genomes (KEGG) pathway enrichment analysis using the WebGestalt database (http://www.webgestalt.org) to confirm the major biochemical metabolic pathways and signaling pathways of the target gene [18].

### Study subjects

Following procedures in previous studies [14, 15], we collected CRC and adjacent normal tissue samples from 169 patients with CRC (95 males and 74 females; mean age 57 years, range 44 to 72 years), obtained by surgical resection at the Harbin Medical University Tumour Hospital. Patients had no pre-surgical treatments, such as chemotherapy, radiotherapy or biotherapy or other therapies. A follow-up monitoring was conducted on all patients until April 30, 2016. The resected primary tumor specimen was evaluated using the tumor node metastasis (TNM) staging system (American Joint Committee on Cancer (AJCC), version 6.0) [19]. The degree of differentiation of the tumor was evaluated according to the classification of tumors from the World Health Organization (WHO); accordingly, the tumors were categorized into undifferentiated, poorly differentiated, moderately differentiated, and well differentiated tumors. The pathological diagnosis [20] was confirmed by two senior pathologists.

### Reverse transcription quantitative polymerase chain reaction (RT-qPCR)

The total RNA of tissues was extracted according to the instructions provided in the Trizol kit (NO. 15596–018; Invitrogen Inc., Carlsbad, CA, USA). We then undertook reverse transcription of the extracted RNA to complementary DNA (cDNA), again following manufacturer’s instructions of the reagent kit (Thermo Fisher Scientific Inc., Waltham, MA, USA) using a two-step method. The obtained cDNA was stored at − 80 °C until use in the subsequent experiments. RT-qPCR was conducted using the TaqMan probe method, following the instructions of the reagent kit (KR011A1; Bejing Puyihua Science and Technology Co., Ltd., Bejing, China). The primer sequences are shown in Table 1. Glyceraldehyde-3-phosphate dehydrogenase (GAPDH) was used as an internal reference of gene expression. The relative mRNA expression of the target gene was expressed as 2^-ΔΔCt^. The experiments were repeated three times [21]. This method was also used for the mRNA detection in the subsequent cell experiments.

### Immunohistochemistry

The tissues were fixed in 10% neutral formalin, dehydrated, embedded in paraffin, cut into 5 μm serial sections, and dehydrated. The tissue sections were then treated with 3% hydrogen peroxide at room temperature for 10 min in order to block endogenous peroxidase activity, after which the sections were blocked with normal nonimmunone serum for 10 min. The sections were then incubated with primary rabbit polyclonal antibodies against phosphorylated (p)-STAT5 (Tyr694/699, SC-81524, Santa Cruz Biotechnology, Inc., Santa Cruz, CA, USA) at a dilution of 1:100, p-STAT6 (Tyr64, SC-136019, Santa Cruz Biotechnology, Inc., Santa Cruz, CA, USA) at a dilution of 1:200, and Cyclin D1 (CCND1) at 4 °C overnight. Sections were then incubated with horseradish peroxidase-labeled secondary antibody goat anti-rabbit immunoglobulin G (IgG) diluted to 1:500 at 37 °C for 20 min, followed by incubation with 50 μl of streptavidin peroxidase solution at room temperature for 10 min and final development with diaminobenzidine (DAB) solution. After counterstaining with hematoxylin, all sections were dehydrated, cleared, and mounted for microscopic examination, with phosphate buffered saline (PBS) solution as negative control (NC). We evaluated the protein positive expression as follows: five visual fields of tumor area at high magnification (× 400) were randomly selected from each section. The positive expression rate was determined according to the percentage of positive cells to the total number of cells. The density of positive cells was determined according to the semi-quantitative assessment with reference to the percentage ratio of positive cells. In the event that the ratio was less than 15%, the protein expression was deemed negative (−); when the ratio ranged from 15 to 25%, the protein expression was weakly positive (+); 25 to 50 was moderately positive (++), ratio 50–75% was strongly positive (+++), and a ratio exceeding 75% indicated that protein expression was significantly and strongly positive (++++). The sections were classified into two groups according to the staining results: sections with a ratio of p-STAT5 and p-STAT6 positive cells < 15% were assigned to the negative group, and sections with a corresponding ratio of exceeding 15% were assigned to the positive group. In the case of CCND1, sections with a ratio of CCND1 positive cells less than 5% were deemed negative, and sections with the ratio exceeding 5% were deemed positive.

### Cell culture

We used the following cell lines provided by the Heilongjiang Tumor Institute: (1) human CPC cell lines of RKO, poorly-differentiated colon cancer cell line, (2) LOVO, a cell line first isolated in 1971 from a segment of metastatic tumor nodule in a 56-year-old Caucasian male colon cancer patient, (3) SW620, which was isolated from a lymph node from a 51-year-old Caucasian patient with blood group A, Rh+), (4) SW480, isolated from a 50-year-old Caucasian male with primary colon cancer, and (5) HCT116, which has a nearly diploid stemline chromosome number, with a modal number of 45 chromosomes (62%) and polyploids occurring in 6.8% of cells. Cells were incubated at 37 °C with 5% CO_2_ in Roswell Park Memorial Institute (RPMI) 1640 culture medium containing 15% fetal bovine serum (FBS) (Santa Cruz Biotechnology, Inc., Santa Cruz, CA, USA).

### Establishment of RNA interference (RNAi) vector

The sequences of RP11-468E2.5 small interference RNA (siRNA) were designed using the Ambion siRNA design software (Austin, TX, USA), with the TTCAAGAGA sequence in the loop structure, and employing the GP-Supersilencing Vector. The lentivirus-based packaging system (L110424; Shanghai Beinuo Biotechnology Co., Ltd., Shanghai, China) consisted of the following four kinds of plasmids: pRsv-REV, p MDlg-pRRE, pMD2G, and an interfering plasmid.

### Cell grouping and transfection

HCT116 and SW480 cells in the logarithmic growth phase were seeded into a six-well culture plates at a cell density of 4 × 10^5^ cells per well. After cell confluence reached 80 to 90%, we conducted X-tremeGENE siRNA transfection. The transfection reagents were mixed and left to stand for 20 min before transfer into the six-well culture plates. After transfection, cells were incubated at 37 °C with 5% CO_2_ and saturated humidity for 48 h. After replacing the culture medium, the cells were incubated for 24 to 48 h. Each cell line was assigned to the blank (without any transfection), NC (transfected with NC sequence for siRNA against RP11-468E2.5), si-lncRNA (transfected with siRNA sequence against RP11-468E2.5), AG490 (treated with AG490, the inhibitor of the JAK/STAT signaling pathway), and AG490 + si-lncRNA groups (co-treated with siRNA sequence against RP11-468E2.5 and AG490).

### Western blot analysis

The collected cells were treated with 100 μl of radioimmunoprecipitation assay (RIPA) lysis buffer (R0020; Beijing Solarbio Science & Technology Co., Ltd., Beijing, China) containing 1 mM phenylmethylsulphonyl fluoride (PMSF) to extract the proteins, after which the protein concentration was measured using a bicinchoninic acid (BCA) kit (AR0146; Wuhan Boster Biological Technology Ltd., Wuhan, Hubei, China). The proteins were then separated by 10% polyacrylamide gel electrophoresis (PAGE), and transferred onto a polyvinylidene difluoride (PVDF) membrane (P2438; Sigma-Aldrich Chemical Company, St Louis MO, USA). Next, the membrane was blocked with addition of 5% bovine serum albumin (BSA), followed by incubation at room temperature for 1 h. The membranes were then incubated with diluted primary antibodies at 4 °C overnight: JAK2 (ab108596), STAT3 (ab68153), STAT5 (ab16276), p-STAT5 (ab98338), STAT6 (ab32520), p-STAT6 (ab28829), CCND1 (ab16663), B-cell lymphoma 2 (Bcl-2) (ab32124), P21 (ab109520), and P27 (ab171091). All above antibodies were purchased from Abcam Inc. (Cambridge, MA, USA). Next morning, the membranes were washed three times (5 min each) in Tris-buffered saline Tween-20 (TBST), and then incubated at room temperature for 1 h with the corresponding secondary anti-rabbit antibody (1:2000; ab6721; Abcam Inc., Cambridge, MA, USA). After three rinses (5 min each) in TBST, the immunocomplexes on the membrane were visualized using enhanced chemiluminescence (ECL) reagent and Gel Imager (Gel Doc EZ Imager; Bio-Rad Laboratories, Hercules, CA, USA). GAPDH (1:10000; ab181602; Abcam Inc., Cambridge, MA, USA) was used as an internal reference, with the relative expression of protein was expressed as the gray value of the target protein to that of the internal reference. Image J software was used to analyze the gray value intensity of the bands of target proteins.

### RNA pull-down assay

Cells were probed for RNA expression with RP11-468E2.5 labeled by 50 nM biotin, collected, and washed using PBS. The cells were incubated in lysis buffer (Ambion, Austin, TX, USA) for 10 min, followed by the addition of streptavidin-coated agarose bead (Invitrogen, Carlsbad, CA, USA) for further culture at room temperature for 1 hr. After washing the beads with PBS, proteins were harvested and analyzed by western blot analysis.

### RNA binding protein immunoprecipitation (RIP)

The binding interactions of RP11-468E2.5 with STAT5 or STAT6 protein were detected using the RIP kit (Millipore Inc., Bedford, MA, USA). Briefly, after a pre-cooled PBS wash, the supernatant was discarded. Then, cells were lysed with lysis buffer and centrifuged at 4 °C and 12,000 g for 10 min. A small amount of supernatant was taken as an input, and the remainder was co-precipitated with antibodies. Specifically, the beads were washed in PBS and resuspended in 100 μl RIP wash buffer. To each group of samples, we added 5 μg diluted antibodies against STAT5 (1:100; ab36153; Abcam Inc., Shanghai, China), STAT6 (1:80; ab32520; Abcam Inc., Shanghai, China), or IgG (1:1000; ab172730; Abcam Inc., Shanghai, China). Here, the IgG group served as NC. The bead-antibody complex was then washed and resuspended in 900 μl RIP wash buffer, followed by incubation with 100 μl cell extract overnight at 4 °C. The samples were then placed on a permanent magnetic base to collect the magnetic bead-protein complexes. RNA was extracted after digestion of the samples and of the total input sample with proteinase K prior to further PCR quantitation.

### 3-(4,5-dimethylthiazol-2-yl)-2,5-diphenyl-tetrazolium bromide (MTT) assay

When cell confluence reached approximately 80%, HCT116 and SW480 cells were washed twice with PBS and detached with 0.25% trypsin to prepare a single cell suspension. Cells were counted and seeded in 96-well plates, with 200 μl volumes containing 3 to 6 × 10^3^ cells per well. Six wells were established for each group, and after a 48-h incubation, 20 μl MTT solution was added to each well (A2776-1 g; 5 mg/ml; Shanghai Shifeng Biological Technology Co., Ltd., Shanghai, China) and incubated for an additional 4 h. The culture medium was discarded, and 150 μl dimethylsulfoxide (DMSO) was added to each well, followed by gentle agitation for 10 min. An enzyme-linked immunosorbent assay (ELISA) reader was used to measure the optical density (OD) values at a wavelength of 490 nm in each well at 12, 24 and 48 h. Then a curve of cell viability was plotted, with triplicate repetitions of the experiment.

### Propidium iodide (PI) staining

Cells were collected and centrifuged, and the supernatant was discarded. Cells were then recentrifuged at 1200 rpm at 4 °C. After being rinsed twice with cooled PBS, the cells were fixed with pre-cooled 70% ethanol at 4 °C overnight. The following day, cells were collected, centrifuged, washed with pre-cooled PBS, and re-suspended. Following the re-suspension, 100 μl portions were added and mixed with RNase to obtain a final concentration of 1 mg/ml, and left in a water bath at 3 °C for 30 min. After the addition of PI staining solution to obtain a final concentration of 50 μg/ml, cells were stained at 4 °C, in subdued light for 40 min and rinsed with PBS. PI was detected at a wavelength > 575 nm and the percentage of cells at each stage of the cycle was calculated.

### Annexin V-fluorescein isothiocyanate (FITC)/PI double staining

An annexin V-FITC/PI double staining reagent kit (556,547; Shanghai Solja Technology Co., Ltd., Shanghai, China) was used to analyze the rate of cell apoptosis. Cells from all groups were centrifuged at 2000 rpm for 5 min, and then resuspended and centrifuged again at 200 rpm for 5 to 10 min. Cells were washed suspended in 300 μl diluted binding buffer and then incubated with 5 μl Annexin V-FITC in subdued light at room temperature for 15 min. Cells were then placed in an ice bath in the dark for 5 min along with 5 μl PI staining solution prior to conducting flow cytometry analysis. Flow cytometry was conducted to determine FITC concentration at excitation wavelengths of 480 and 530 nm and PI at an excitation wavelength > 575 nm.

### Immunofluorescence assay

The cell slides were treated with polylysine at room temperature for 1 h and rinsed three times with sterile water (5 min each), followed by a 30-min ultraviolet irradiation period. The slides were placed into 12-well plates, which were washed twice with PBS, whereupon the wells were seeded with CRC cell lines (HCT116 and SW480). Allowing 48 h for adherence of the cells to the chamber walls, cells were fixed in 4% paraformaldehyde for 20 min at room temperature and immersed three times (5 min each) in pre-cooled PBS. Next, cells were blocked with the addition of 3% BSA/phosphate buffered saline with Tween-20 (PBST) at room temperature for 1 h, incubated with 3% BSA/PBST-diluted primary antibody (p-STAT5, 1:200; p-STAT6, 1:200) at 4 °C overnight, and next morning rinsed three times with PBS. Subsequently, cells were incubated with 3% BSA/PBST-diluted secondary antibody in subdued light for 1 h, rinsed three times with pre-cooled PBS, and then incubated with 0.1 μg/ml 4′6-diamidino-2-phenylindole (DAPI) for 1 min. After being rinsed three times in ice cold PBS, cells were sealed and imaged with a microscope.

### In vivo tumor formation assay

A total of 30 BALB/c nude mice (aged from 4 to 6 weeks; weighed 17 g to 20 g) were purchased from SLAC Laboratory Animal Co., Ltd. (Changsha, China) and housed in a specific pathogen-free environment. The nude mice were then subcutaneously injected with 2 × 10^7^ cells of the types mentioned above. Afterwards, the mice were euthanized, and the tumors extracted, photographed, and measured with a vernier caliper. Tumor volume was calculated with the formula (a × b^2^)/2, where a represented the shorter axis and b the longer axis. We then plotted the mean volume at each time point (*n* = 6 in each group).

### Statistical analysis

SPSS 21.0 software (IBM Corp., Armonk, NY, USA) was applied for data analyses. The measurement data were expressed as mean ± standard deviation. One-way analysis of variance (ANOVA) was used for multiple group comparisons. Comparisons between two groups were conducted by *t*-test. *p* < 0.05 was considered statistically significant.

## Results

### LncRNA expression profiles in CRC

**Fig. 1:**
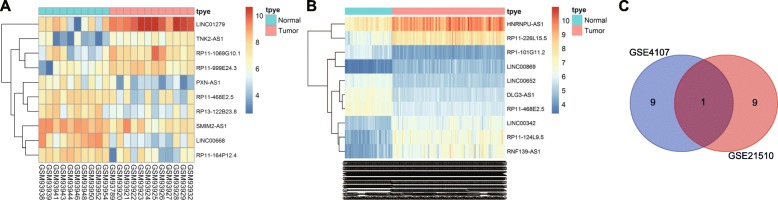
Microarray-based gene expression profiling of CRC identified RP11-468E2.5 for the study (**a**: GSE4107; **b**: GSE21510); the abscissa represents the sample number, and the ordinate represents the DEGs; the histogram at the upper right refers to color gradation; each rectangle corresponds to a sample expression value; red indicates higher expression and green indicates lower expression. **c** Intersection of the top 10 lncRNAs with the most differential expression in microarray data. CRC, colorectal cancer; DEGs, differentially expressed genes

To search for differentially expressed lncRNAs in CRC, two lncRNA-related microarray data entries were retrieved from the GEO database for CRC. The GSE4107 is a dataset for early onset CRC, which includes 22 samples, ten of which are normal and 12 are CRC. Comparatively, GSE21510 consists of a total of 148 samples, which includes 25 normal and 123 CRC samples. Microarray data GSE4107 and GSE21510 related to CRC were analyzed using R language, and the top ten lncRNAs with the most differential expression in both microarrays were profiled (Fig. 1a~b). The analysis of microarray data showed that among the top ten lncRNAs, only RP11-468E2.5 had low expression levels in CRC (Fig. 1c). Therefore, we used RP11-468E2.5 for this study.

### STAT5 and STAT6 are target genes of RP11-468E2.5

With the microarray-based gene expression profiled, the next step was to examine the target genes of RP11-468E2.5. To do so, the MEM website was used to assist in predicting the target genes of lncRNA. The results showed that STAT5 and STAT6 were target genes of RP11-468E2.5, such that both proteins contributed to regulation of the JAK/STAT signaling pathway (Table 2).

### RP11-468E2.5 is negatively correlated with the JAK/STAT signaling pathway-related genes

**Fig. 2:**
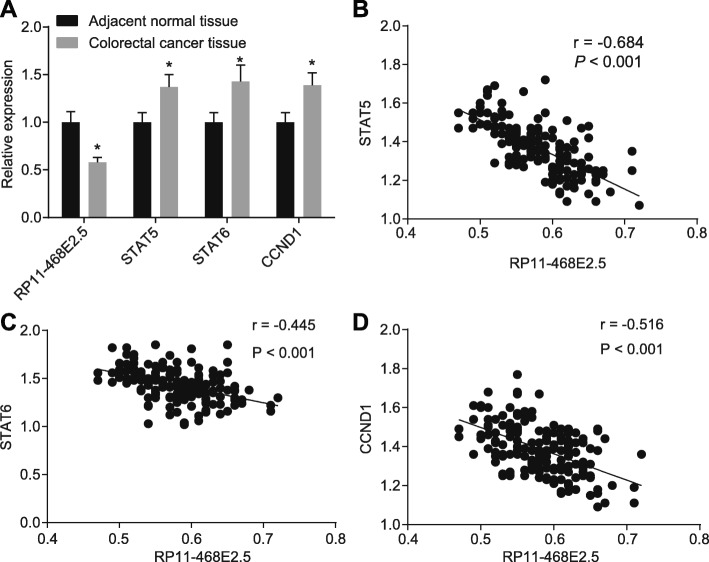
RP11-468E2.5 and the JAK/STAT signaling pathway-related genes are negatively correlated in CRC tissues. A, The expression of RP11-468E2.5, STAT5, STAT6 and CCND1 detected by RT-qPCR. B, The correlation between RP11-468E2.5 with STAT5, STAT6 and CCND1 using Pearson correlation analysis. JAK, janus kinase; lncRNA, long non-coding RNA; RT-qPCR, reverse transcription quantitative polymerase chain reaction; STAT5, signal transducer and activator of transcription-5; STAT6, signal transducer and activator of transcription-6; CCND1, Cyclin D1; CRC, colorectal cancer. * *p* < 0.05, compared with the adjacent normal tissues

RT-qPCR was employed to determine expression of RP11-468E2.5 and the JAK/STAT signaling pathway-related genes in CRC tissues. When compared to the adjacent normal tissues, CRC tissues showed lower expression of RP11-468E2.5 and higher mRNA expression of STAT5, STAT6 and CCND1 (all *p* < 0.05) (Fig. 2a). Pearson correlation analysis suggested that there was a negative correlation between the expression of RP11-468E2.5 and that of STAT5, STAT6, and CCND1 (Fig. 2b-d).

### CRC tissues show increased positive expression of p-STAT5, p-STAT6 and CCND1 proteins

**Fig. 3:**
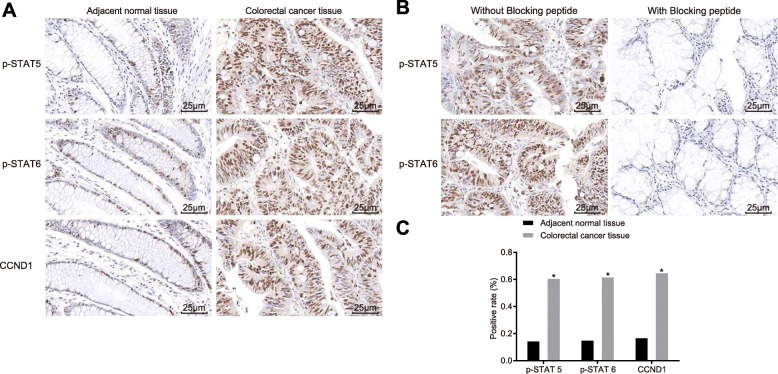
Increased positive expression of p-STAT5, p-STAT6 and CCND1 proteins is evident in CRC tissues. **a** The expression of p-STAT5, p-STAT6 and CCND1 in CRC tissue detected by immunohistochemistry (× 400). **b** Antibody labeling conditions following p-STAT5 and p-STAT6, with blocking peptide treatment in immunohistochemistry. **c** Quantitative analysis for positive expression of p-STAT5, p-STAT6 and CCND1. p-STAT5, p-signal transducer and activator of transcription-5; p-STAT6, p-signal transducer and activator of transcription-6; CRC, colorectal cancer; NC, negative control

We used immunohistochemistry (SP method) to measure the extent of STAT5/STAT6 phosphorylation and the expression of CCND1 proteins in CRC tissues and adjacent normal tissues. The results here showed a distinct cell morphology, with the extent of STAT5/STAT6 phosphorylation and the expression of CCND1 presenting as brownish-yellow and brown pellets in the nucleus or cytoplasm. In comparison to adjacent normal tissues, the positive expression of p-STAT5, p-STAT6 and CCND1 proteins was higher in CRC tissues (all *p* < 0.05) (Fig. 3a). We stained the proteins using normal goat serum instead of the primary antibody, which served as NC. The specific staining of p-STAT5 and p-STAT6 was negative after blocking peptide treatment, indicating high specificity of the two antibodies (Fig. 3b).

In CRC tissues, the positive expression rate of p-STAT5 was 47.9% (81/169), and that of CCND1 was 56.2% (95/169). Pearson correlation analysis showed a positive correlation between the expression of the two proteins (r = 0.727, *p* < 0.001). There was also a positive correlation between the expression of p-STAT6 and CCND1. The positive expression rate of p-STAT6 was 50.9% (86/169) and that of CCND1 was 56.2% (95/169) (r = 0.731, *p* < 0.001) (Table 3).

### The extent of STAT5/STAT6 phosphorylation is related to the tumor infiltration depth of patients with CRC

Following the CRC tissue analysis, we made a correlation analysis between p-STAT5, p-STAT6 and CCND1 protein expression and the clinicopathological features in our database of patients with CRC. Among the 169 patients with CRC, there were 81 cases with a positive expression of p-STAT5 (47.93%), and 88 cases of negative expression of p-STAT5 (52.07%). There were furthermore 86 cases with positive expression of p-STAT6 (50.88%) and 83 cases with negative expression of p-STAT6 (49.11%), and 95 cases with positive expression of CCND1 (56.21%) and 74 cases with a negative expression of CCND1 (43.78%) (Table 3 and 4). A subgroup analysis of p-STAT5 and p-STAT6 protein expression was performed, along with clinicopathological features in the CRC patients, i.e. age, gender, tumor size, tumor site, differentiation degree, infiltration depth, lymphatic metastasis and distant metastasis. The results of this analysis showed a positive correlation between p-STAT5 and p-STAT6 protein expression and infiltration depth (T stage) (*p* = 0.002). There was no correlation of the proteins with the other covariates, i.e. age, gender, tumor size, tumor site, differentiation degree, lymphatic metastasis and distant metastasis (all *p* > 0.05). We found that CCND1 protein expression was not correlated with the age, gender, tumor size, tumor site, differentiation degree, infiltration depth (T stage), lymphatic metastasis or distant metastasis (all *p* > 0.05) (Table 4).

Furthermore, we conducted subgroup analysis on RP11-468E2.5 expression and the clinicopathological features as above among the 169 patients with CRC (Table 4). The results demonstrated that RP11-468E2.5 expression was correlated positively with T stage (*p* = 0.039) and distant metastasis (*p* = 0.011) but had no significant correlation with age, gender, tumor size, site, differentiation or lymph node metastasis (*p* > 0.05). Therefore, in compiling the above results, we infer that the tumor infiltration depth of CRC patients must correlate positively with the extent of STAT5/STAT6 phosphorylation.

### Highly expressed STAT5 and STAT6 are found in LOVO, SW620, SW480 and HCT116 cell lines

**Fig. 4:**
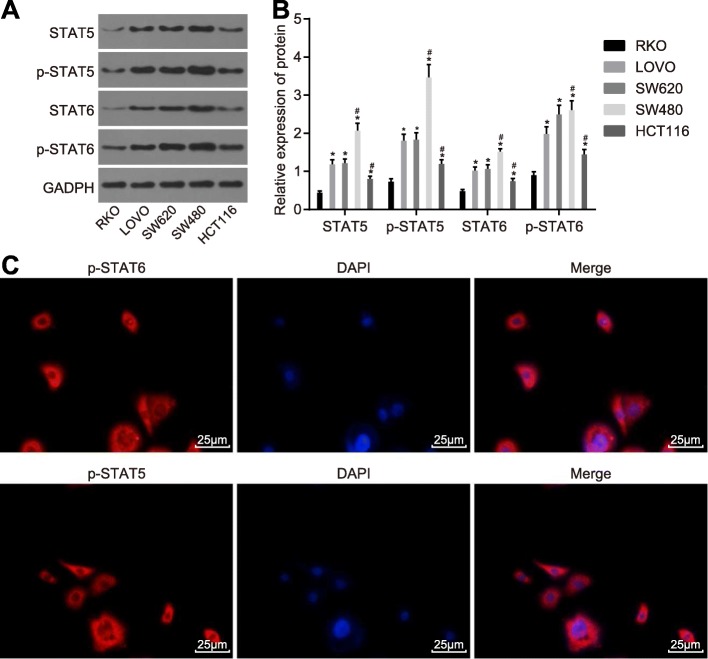
Increased protein expression of STAT5 and STAT6 is found in LOVO, SW620, SW480 and HCT116 cell lines. A and B, Western blot analysis of STAT5, p-STAT5 STAT6, p-STAT6 and GAPDH proteins in different cell lines. C, Subcellular localization of p-STAT5/6 in CRC cells detected by immunofluorescence assay. p-STAT6, p-signal transducer and activator of transcription-6; STAT6, signal transducer and activator of transcription-6; p-STAT5, p-signal transducer and activator of transcription-5; STAT5, signal transducer and activator of transcription-5; GAPDH: glyceraldehyde-3-phosphate dehydrogenase. * *p* < 0.05, compared with RKO cell line; # *p* < 0.05, compared with LOVO cell line

We used Western blot analysis to measure protein expression of STAT5 and STAT6 in CRC cell lines (RKO, LOVO, SW620, SW480 and HCT116). As depicted in Fig. 4, the protein expression of STAT5, STAT6 and the extent of STAT5 and STAT6 phosphorylation in LOVO, SW620, SW480 and HCT116 cell lines were higher when compared to that in RKO cell lines (all *p* < 0.05). Among the tumor cells, the SW480 cell lines in particular showed the highest expression of all proteins (all *p* < 0.05). Therefore, we selected HCT116 and SW480 cell lines for subsequent experiments due to their generally lower metastasis and higher protein expression, respectively; we did not proceed with SW620 and LOVO cell lines because of their high metastasis. In addition, the results of subcellular localization of p-STAT5/6 in CRC cells detected by immunofluorescence assay indicated cytoplasmic retention in CRC cells, which was in accordance with immunohistochemistry results.

### Effects of RP11-468E2.5 on mRNA expression of the JAK/STAT signaling pathway- and apoptosis-related genes in HCT116 and SW480 cells

**Fig. 5:**
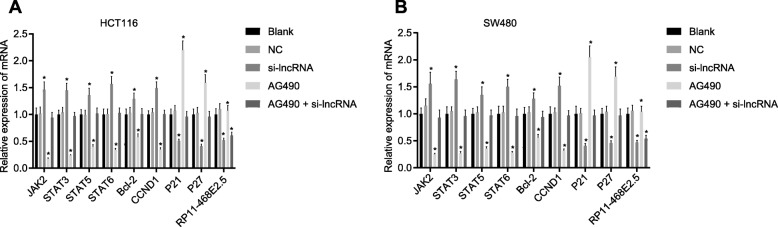
Effect of RP11-468E2.5 on the mRNA expression of JAK/STAT signaling pathway- and apoptosis-related genes in HCT116 and SW480 cells. **a** RP11-468E2.5 expression and mRNA expression of the JAK/STAT signaling pathway-related genes and apoptosis-related genes determined by RT-qPCR in HCT116 cells. **b** RP11-468E2.5 expression and mRNA expression of the JAK/STAT signaling pathway- and apoptosis-related genes determined by RT-qPCR in SW480 cells; RT-qPCR, reverse transcription quantitative polymerase chain reaction; NC, negative control; JAK1, janus kinase 1; STAT3, signal transducer and activator of transcription-3; STAT5, signal transducer and activator of transcription-5; STAT6, signal transducer and activator of transcription-6; Bcl-2, B-cell leukemia/lymphoma 2; CCND1, Cyclin D1. * *p* < 0.05, compared with the blank group

We applied RT-qPCR to determine the expression of RP11-468E2.5 and mRNA expression of JAK/STAT signaling pathway- and apoptosis-related genes in HCT116 and SW480 cells after transfection. The results (Fig. 5) showed no significant differences in mRNA expression of JAK1, STAT3, STAT5, STAT6, CCND1, Bcl-2, P21 and P27 in HCT116 and SW480 cells in the blank, NC, and AG490 + si-lncRNA groups (all *p* > 0.05). When compared to the blank group, we found that the AG490 + si-lncRNA group showed lower RP11-468E2.5 expression (*p* < 0.05). Comparatively, the si-lncRNA group showed much higher mRNA expression of JAK2, STAT3, STAT5, STAT6, CCND1 and Bcl-2, but relatively lower mRNA expression of P21 and P27, and RP11-468E2.5 expression, while the expression pattern tended to be opposite in the AG490 group (all *p* < 0.05). From these findings, we inferred that up-regulated RP11-468E2.5 could decrease the mRNA expression of JAK2, STAT3, STAT5, STAT6, CCND1 and Bcl-2, but increase that of P21 and P27.

### Effects of RP11-468E2.5 on protein expression of the JAK/STAT signaling pathway- and apoptosis-related factors in HCT116 and SW480 cells

**Fig. 6:**
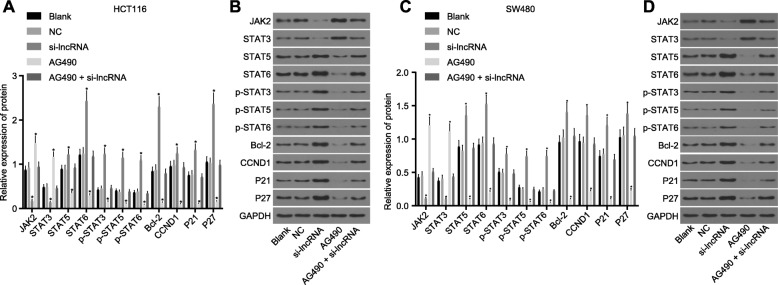
Effects of RP11-468E2.5 on the protein expression of JAK/STAT signaling pathway- and apoptosis-related genes in HCT116 and SW480 cells. **a** and **b** Western blot analysis of the JAK/STAT signaling pathway- and apoptosis-related proteins in HCT116 cells. **c** and **d** Western blot analysis of the JAK/STAT signaling pathway- and apoptosis-related proteins in SW480 cells. NC, negative control; JAK1, janus kinase 1; STAT3, signal transducer and activator of transcription-3; STAT5, signal transducer and activator of transcription-5; STAT6, signal transducer and activator of transcription-6; Bcl-2, B-cell leukemia/lymphoma 2. * *p* < 0.05, compared with the blank group

We used a Western blot analysis to determine the protein expression of the JAK/STAT signaling pathway- and apoptosis-related genes after transfection. The results (Fig. 6) showed no significant differences in protein expression of JAK1, STAT3, STAT5, STAT6, CCND1, Bcl-2, P21 and P27 as well as extent of STAT6, STAT3 and STAT5 phosphorylation in HCT116 and SW480 cells in the blank, NC, and AG490 + si-lncRNA groups (all *p* > 0.05). When compared to the blank group, there was an upward trend in the protein expression of JAK2, STAT3, STAT5, STAT6, CCND1 and Bcl-2, while a downward trend in P21 and P27 protein expression in the si-lncRNA group (*p* < 0.05). Furthermore, the AG490 group exhibited an opposite trend as compared with the si-lncRNA group (all *p* < 0.05). These findings indicated that up-regulated RP11-468E2.5 could decrease the protein expression of JAK2, STAT3, STAT5, STAT6, CCND1 and Bcl-2 along with extent of STAT6, STAT3 and STAT5 phosphorylation, but increase that of P21 and P27.

### An interaction exists among RP11-468E2.5, STAT5 and STAT6

**Fig. 7:**
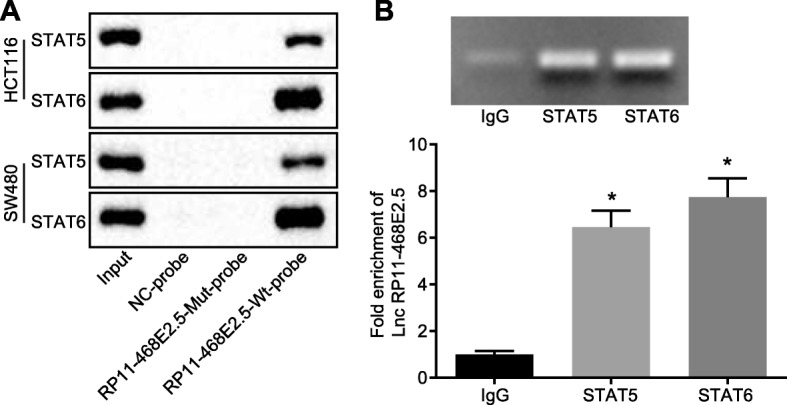
RP11-468E2.5 interacts with STAT5 and STAT6. **a** Interaction among RP11-468E2.5, STAT5 and STAT6 detected by RNA pull-down assay. **b** Interaction among RP11-468E2.5, STAT5 and STAT6 verified using RIP assay. STAT5, signal transducer and activator of transcription-5; STAT6, signal transducer and activator of transcription-6. * *p* < 0.05, compared with IgG group

To further identify the possible interaction among RP11-468E2.5, STAT5 and STAT6, we performed RNA pull-down and RIP assays. Based on the RNA pull-down results, STAT5 and STAT6 were enriched in RP11-468E2.5 wild type (WT) animals in contrast to RP11-468E2.5 mutant type (MUT) and NC (Fig. 7a). Meanwhile, according to RIP detection of the combination of RP11-468E2.5, STAT5, and STAT6 proteins (Fig. 7b), there was increased binding of RP11-468E2.5 to STAT5 and STAT6, as compared to IgG (*p* < 0.05). These results revealed an interaction between RP11-468E2.5, STAT5 and STAT6.

### Up-regulation of RP11-468E2.5 inhibits proliferation of HCT116 and SW480 cells

**Fig. 8:**
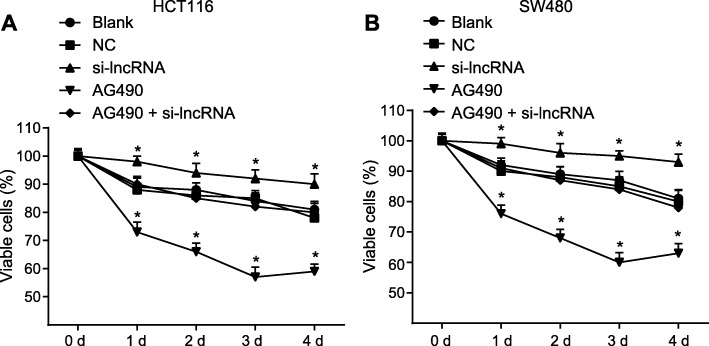
Up-regulation of RP11-468E2.5 inhibits proliferation of HCT116 and SW480 cells. **a** Cell proliferation of HCT116 cells determined by MTT assay. **b** Cell proliferation of SW480 cells determined by MTT assay. MTT, 3-(4,5-dimethylthiazol-2-yl)-2,5-diphenyl-tetrazolium bromide; NC, negative control. * *p* < 0.05, compared with the blank group

With the effects of RP11-468E2.5 on protein expression of the JAK/STAT signaling pathway now fully examined, the next step in this project was to observe the effect of RP11-468E2.5 up-regulation on proliferation of HCT116 and SW480 cells. To this end we employed an MTT assay to detect cell proliferation. The results suggested that, relative to the blank group, the si-lncRNA group showed an elevated cell vitality (*p* < 0.05), whereas the AG490 group exhibited a reduced vitality (*p* < 0.05). There were no differences in cell vitality among the NC, AG490 + si-lncRNA and blank groups (all *p* > 0.05) (Fig. 8). Therefore, we concluded that increased RP11-468E2.5 repressed the proliferation ability of HCT116 and SW480 cells.

### Up-regulation of RP11-468E2.5 increases HCT116 and SW480 cells arrested in G1/G0 phase

**Fig. 9:**
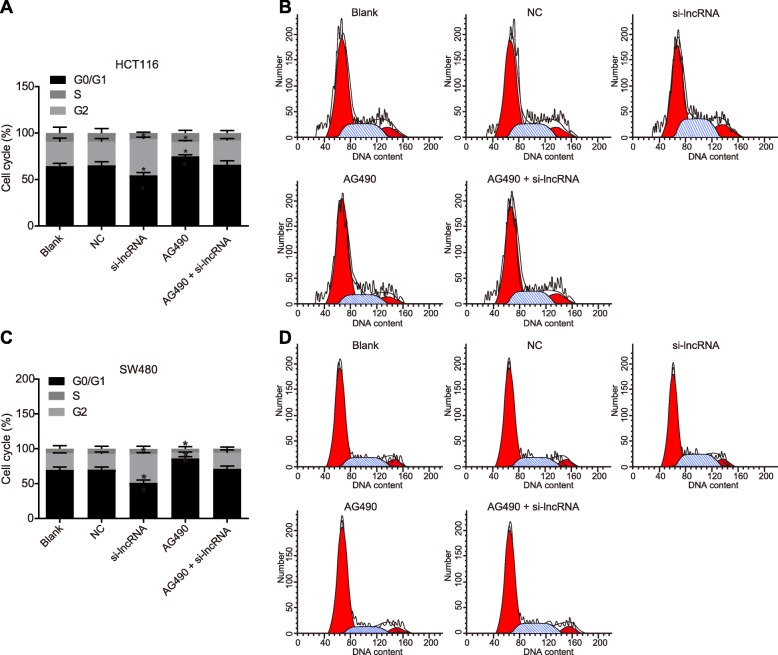
Up-regulation of RP11-468E2.5 increases HCT116 and SW480 cells arrested in G1/G0 phase. **a** Quantitative analysis for cell cycle of HCT116 cells in all groups. **b** Cell cycle distribution of HCT116 cells detected by flow cytometry. **c** Quantitative analysis for cell cycle of SW480 cells in all groups. **d** Cell cycle distribution of SW480 cells detected by flow cytometry. NC, negative control; PI, propidium iodide. * *p* < 0.05, compared with the blank group

Cell cycle distribution of HCT116 and SW480 cells in each group was assessed by PI staining. As shown in Fig. 9, there was no difference in cell cycle distribution among the blank, NC and AG490 + si-lncRNA groups (all *p* > 0.05). The proliferation ability of HCT116 and SW480 cells in the AG490 group was relatively attenuated, with more cells arrested in the G1/G0 phase (*p* < 0.05). Furthermore, there were fewer cells arrested at S phase and G2 phase when compared to the blank group (all *p* < 0.05). In the si-lncRNA group, we found fewer cells arrested at the G1/G0 phase, but increased numbers arrested at S phase and G2 phase (all *p* < 0.05). These results indicated that up-regulated RP11-468E2.5 arrested cells in the G1/G0 phase.

### Up-regulation of RP11-468E2.5 promotes apoptosis of HCT116 and SW480 cells

**Fig. 10:**
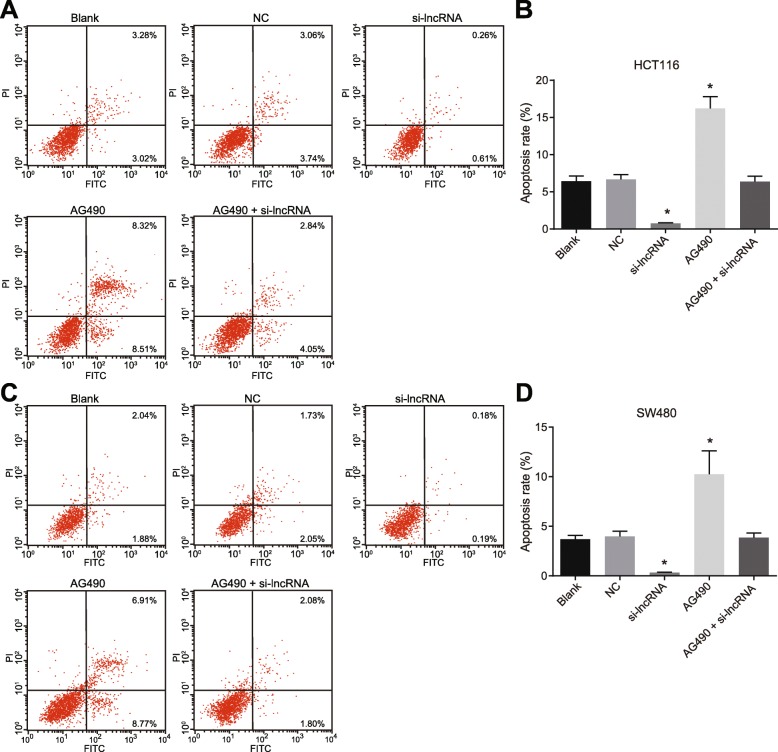
Up-regulation of RP11-468E2.5 promotes apoptosis of HCT116 and SW480 cells. **a** Cell apoptosis in HCT116 cells detected by flow cytometry. **b** Quantitative analysis for cell apoptosis rate in HCT116 cells in all groups. **c** Cell apoptosis in SW480 cells detected by flow cytometry. **d** Quantitative analysis for cell apoptosis rate in SW480 cells in all groups. NC, negative control; FITC; fluorescein isothiocyanate; PI, propidium iodide. * *p* < 0.05, compared with the blank group

To determine the effects of up-regulation of RP11-468E2.5 on the apoptosis rate of HCT116 and SW480 cells in each group, we performed Annexin V-FITC/PI double staining. There was no significant difference in the apoptosis rates during the early and late stages among the blank, NC and AG490 + si-lncRNA groups (all *p* > 0.05). The AG490 group exhibited a higher apoptosis rate during the early and late stages when compared to the blank group (all *p* < 0.05), while the si-lncRNA group showed a lower apoptosis rate during both early and late stages (all *p* < 0.05) (Fig. 10). Therefore, we concluded that up-regulated RP11-468E2.5 could enhance the apoptosis of HCT116 and SW480 cells.

### RP11-468E2.5 silencing stimulates the growth of tumors

**Fig. 11:**
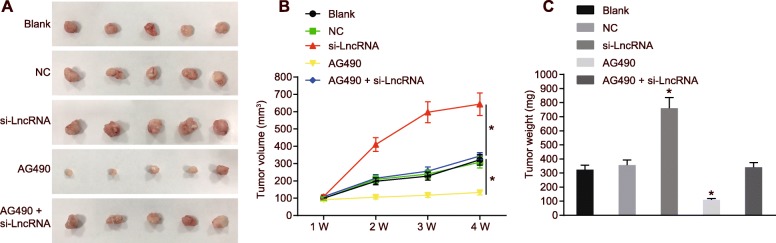
Knockdown of RP11-468E2.5 drives tumor growth. **a** and **b**, Xenograft tumors and quantitative analysis of tumor volume and mass in HCT116 cells after si-lncRNA and AG490 + si-lncRNA treatment. **c** and **d**, Xenograft tumors and quantitative analysis of tumor volume and mass in SW480 cells after si-lncRNA and AG490 + si-lncRNA treatment. *n* = 6. * *p* < 0.05, compared with the blank group

Finally, we conducted xenograft tumor assay to investigate the effect of RP11-468E2.5 on tumor growth in vivo. Here, results suggested that the tumors treated with siRNA against RP11-468E2.5 had the greatest volume and weight, while tumors with AG490 treatment had the smallest (*p* < 0.05). The mice receiving co-treatment of AG490 and RP11-468E2.5 silencing showed no evident difference in tumor growth as compared with that in mice without any treatment (*p >* 0.05) (Fig. 11).

## Discussion

The prognosis of CRC patients remains poor [22]. In recent years, lncRNAs have been found to play a role in controlling gene expression and to regulate cell development, cell migration, cell proliferation and cell apoptosis in carcinomas [23]. In addition to this mechanism, the JAK/STAT signaling pathway also plays an important role in cellular processes and immune function [24]. Therefore, the aim of this study was to investigate the potential effects of RP11-468E2.5 and its target genes, STAT5 and STAT6 on CRC cell biological functions via the JAK/STAT signaling pathway. Our results consistently showed that high RP11-468E2.5 expression inhibits proliferation and promotes apoptosis of CRC cells by negatively regulating STAT5 and STAT6 expression via inactivating the JAK/STAT signaling pathway.

In cancers, a large part of nuclear transcription belongs to lncRNAs, and these molecules are involved in regulating many tumor markers. However, only a small number of lncRNAs have been studied in CRC, and the function of most lncRNAs in CRC remains to be established. Huang et al. indicated that low expression of lncRNA HOXB-AS3 in CRC can encode a small peptide segment, which induces inhibition of PKM shear in its downstream metabolic recombination process, thereby inhibiting tumor growth [25]. SNHGS is an upregulated cytoplasmic lncRNA in CRC, which promotes resistance to chemotherapy with oxaliplatin by preventing STAU1 degradation and stabilizing downstream target genes [26]. In the present study, CRC tissues showed decreased expression of RP11-468E2.5 and increased expression of STAT5, STAT6 and CCND1 compared to adjacent normal tissues. LncRNAs can interact with proteins to participate in regulation of gene expression and therefore aggravate cancer progression [27]. Two members of the STAT protein family, STAT5 and STAT6, have been regarded as critical transcription factors that translocate into the nucleus upon phosphorylation by receptor-associated kinases. Upon activation of those receptors, phosphorylated STAT proteins can regulate nuclear gene expression, and interact with lncRNAs, which can play significant roles in cellular development and tumor progression [28]. In addition to the tumor progression, CCND1 cyclin, cyclin-dependent kinases and cyclin-dependent kinase inhibitors are well-known regulators of cell cycle transition in CRC [29, 30]. There have been indications that the expression of protein coding genes is mediated by their promoter-sharing lncRNA. For example, knockdown via lncRNA by RNAi leads to up-regulation of the expression of protein-coding genes [31]. Previous studies have demonstrated that lncRNA RP11-462C24.1 expression is lower in CRC tissues [32]. Based on this information, we predicted that RP11-468E2.5 is expressed at a low concentration in CRC tissues. When taken together, it was concluded that lower RP11-468E2.5 expression could potentially up-regulate STAT5, STAT6, and CCND1, which made a great difference in facilitating the development and progression of CRC.

Present results show that STAT5 and STAT6 are among the target genes of RP11-468E2.5. When compared to the blank group, the si-lncRNA group showed significantly higher expression of JAK1, STAT3/5/6, CCND1 and Bcl-2, and decreased expression of P21 and P27, while the AG490 group exhibited the opposite trend. Besides, based on our RNA pull-down experiment result, WT RP11-468E2.5 obviously enhanced STAT5 and STAT6 expression in contrast to MUT RP11-468E2.5 or the relevant NC. The RIP assay also verified that RP11-468E2.5 was able to bind to more STAT5 and STAT6 than the IgG control did. Both of these experiments confirm that RP11-468E2.5 interacts with STAT5 and STAT6. Indeed, a previous study has demonstrated that STAT proteins can regulate JAK/STAT signaling pathway related-genes such as STAT5 [33]. According to another previous study, CCND1 and Bcl-2 concentrations are closely related to unfavorable prognosis and low survival rate of patients with pancreatic cancer. The suppression of the STAT3 signaling pathway, which targets both CCND1 and Bcl-2, led to down-regulation of the expression of both proteins, thereby inducing cellular apoptosis in cancer cells [34]. Moreover, lncRNAs participated in the suppression of STAT-3 dephosphorylation in the JAK/STAT signaling pathway, thereby acting as a regulator of cell development and cell growth [35]. The activation of STAT5 also up-regulates downstream target genes including Bcl-xL, cyclinD1/D2 and c-myc, all of which promote esophageal carcinoma cell proliferation, cell survival and immune system evasion [36]. Therefore, we feel confident in inferring that up-regulation of RP11-468E2.5 negatively targeted STAT5/6, and thus played an important role in the expression of the JAK/STAT signaling pathway related-genes and apoptosis related-genes in our CRC cells.

The clinical data in Table 4 showed that the expression of p-STAT5 or p-STAT6 was positively correlated with infiltration depth (T stage) in CRC, but not with other pathological parameters including tumor size. In addition, there was no significant correlation between CCND1 expression and all pathological parameters including tumor size. More importantly, RP11-468E2.5 expression also only correlated with T stage (*p* = 0.039) and distant metastasis (*p* = 0.011), but no with tumor size (*p* > 0.05). STAT5 has been demonstrated to assist IL-23 and thus might only promote the metastasis of CRC [37]. Also, the expression of STAT6 is correlated with lymph node metastasis and distant metastasis (*p* = 0.001 and *p* = 0.016, respectively) [38]. In addition, up-regulation of RP11-468E2.5 results in a significant decrease in the expression of STAT5, STAT6 and CCND1 as well as the extent of STAT5 and STAT6 phosphorylation in CRC cells [39]. These findings suggested that RP11-468E2.5 and p-STAT5 or p-STAT6 might be associated with CRC metastasis and infiltration while exerting little effect on the growth of CRC.

This study also demonstrated that up-regulation of RP11-468E2.5 and inhibition of the JAK/STAT signaling pathway via negatively targeting STAT5 and STAT6 suppressed proliferation and promoted apoptosis of CRC cells. As shown by the nude mouse in vivo tumor formation assay, silencing of RP11-468E2.5 led to the largest tumor growth in vivo, while the treatment with employment of AG490 resulted in slowest tumor growth, proving that down-regulated RP11-468E2.5 boosted CRC cell proliferation and inhibited apoptosis. Indeed, patients with a lower RP11-462C24.1 expression have a worse prognosis along with reduced survival rates, when compared to those with higher RP11-462C24.1 expression levels. These individuals have exhibited reduced cell proliferation, migration and invasion, and promoted apoptosis in CRC cells [32], which is consistent with the present study. Furthermore, the JAK/STAT signaling pathway is proposed to be an indispensable regulator of immune function, cell migration, and apoptosis, where its inhibition has been correlated to the severity of, for example, pancreatic cancer [40]. STAT5 also plays an important role in the maintenance of intracellular organelles as well as regulating cell proliferation, cell differentiation and survival in progenitor B and T cells [28, 41]. Other findings have documented a significant correlation between the development and progression of CRC with components of JAK/STAT signaling pathway such as JAK1, JAK2, and STAT3 [42].

Based on microarray analysis, RP11-468E2.5 was poorly-expressed in CRC samples. The RP11-468E2.5-targeted JAK/STAT signaling pathway could promote CRC cell proliferation and inhibit cell apoptosis. STAT affected cell growth or differentiation by regulating cyclin [43]. There is also a report that STAT5 expression was observed in − 674-CCND1 [44]. Since CCND1, CCND2, p21^WAF/Cip1^, p27^kip^ and anti-apoptotic genes such as Bcl-xl and Bcl-2 are considered to lie downstream to the JAK/STAT signaling pathway [45], their genetic expression was only detected in cellular experiments. Therefore, the role of RP11-468E2.5 in clinical studies remained to be explored and confirmed. Herein, we mainly investigated the effects of RP11-468E2.5 on the JAK/STAT signaling pathway. Inhibition of JAK2 activity by treatment with AG490 significantly altered expression of STAT3/5/6. However, there remains much to be learned about how the specific inhibition of STAT5/6 expression affects CRC cell growth.

This study may draw attention to STAT5/6 as a novel therapeutic target for future CRC treatment. However, we noted some limitations of the present results. For example, the role of p-STAT5/p-STAT6 in tumor cell migration and invasion is still unknown, and further investigations on their function are necessary. Besides, the specific and detailed mechanism by which RP11-468E2.5 negatively targets STAT5 and STAT6 remains largely unclear. As reported in a prior work, lncRNAs could competitively bind to miRNAs to regulate the level of downstream genes [46]. Further research efforts in this direction are strongly recommended.

## Conclusions

In conclusion, this study demonstrated that up-regulated RP11-468E2.5 resulted in inhibited cell proliferation and promoted cell apoptosis in CRC. This effect was obtained via down-regulating STAT5 and STAT6 expression due to suppressing the activation of the JAK/STAT signaling pathway. However, the specific and detailed mechanism by which RP11-468E2.5 negatively targets STAT5 and STAT6 remain unclear. Therefore, further investigation is warranted, aiming ultimately to develop a safer and more efficient method of lncRNA-targeting cancer diagnosis and therapy.

## Data Availability

The datasets generated during the current study are available.

## Abbreviations

AJCC: American Joint Committee on Cancer
ANOVA: Analysis of variance
BCA: Bicinchoninic acid
Bcl-2: B-cell lymphoma 2
BSA: Bovine serum albumin
CCND1: Cyclin D1
cDNA: complementary DNA
CRC: Colorectal cancer
DAB: Diaminobenzidine
DAPI: 4′6-diamidino-2-phenylindole
DMSO: Dimethylsulfoxide
ECL: Enhanced chemiluminescence
ELISA: Enzyme-linked immunosorbent assay
FBS: Fetal bovine serum
FITC: Fluorescein isothiocyanate
GAPDH: glyceraldehyde-3-phosphate dehydrogenase
GEO: Gene Expression Omnibus
IgG: immunoglobulin G
JAK/STAT: Janus kinase-signal transducer and activator of transcription
KEGG: Kyoto Encyclopedia of Genes and Genomes
LncRNAs: Long non-coding RNAs
MTT: 3-(4,5-dimethylthiazol-2-yl)-2,5-diphenyl-tetrazolium bromide
NC: negative control
OD: Optical density
p: phosphorylated
PAGE: Polyacrylamide gel electrophoresis
PBS: Phosphate buffered saline
PBST: Phosphate buffered saline with Tween-20
PI: Propidium iodide
PMSF: Phenylmethylsulphonyl fluoride
PVDF: Polyvinylidene difluoride
RIP: RNA Binding protein immunoprecipitation
RIPA: Radioimmunoprecipitation assay
RPMI: Roswell Park Memorial Institute
RT-qPCR: Reverse transcription quantitative polymerase chain reaction
siRNA: small interference RNA
TBST: Tris-buffered saline Tween-20
TNM: Tumor node metastasis
WHO: World Health Organization
